# Multi-shaped strain soliton networks and moiré-potential-modulated band edge states in twisted bilayer SiC†

**DOI:** 10.1039/d1ra02139g

**Published:** 2021-07-12

**Authors:** Dawei Kang, Zheng-Wei Zuo, Zhaowu Wang, Weiwei Ju

**Affiliations:** School of Physics and Engineering, Henan University of Science and Technology Luoyang 471023 China kdwsdu@163.com; National Laboratory of Solid State Microstructures, Nanjing University Nanjing 210093 China

## Abstract

Tuning the interlayer twist angle provides a new degree of freedom to exploit the potentially excellent properties of two dimensional layered materials. Here we investigate the structural and electronic properties of twisted bilayer SiC under a series of twist angles using first principle calculations. The interplay of interlayer van der Waals interactions and intralayer strain induces dramatic in-plane and out-of-plane displacements. The expansion or contraction of specific stacking domains can be interpreted as the result of the energy minimization rule. By means of order parameter analysis, the triangular or hexagonal strain soliton networks are found to separate adjacent stacking domains. The unique overlapped zigzag atom lines in strain solitons provide a unique characteristic for experimental imaging. The top valence band and bottom conduction band evolve into flat bands with the smallest band width of 4 meV, indicating a potential Mott-insulator phase. The moiré-potential-modulated localization pattern of states in the flat band, which is dependent sensitively on the structure relaxation, controls the flat band width. The moiré-pattern-induced structural and electronic properties of twisted bilayer SiC are promising for application in nanoscale electronic and optical devices.

## Abstract 2

Tuning the interlayer twist angle provides a new degree of freedom to exploit the potentially excellent properties of two dimensional layered materials.

## Graphical abstract

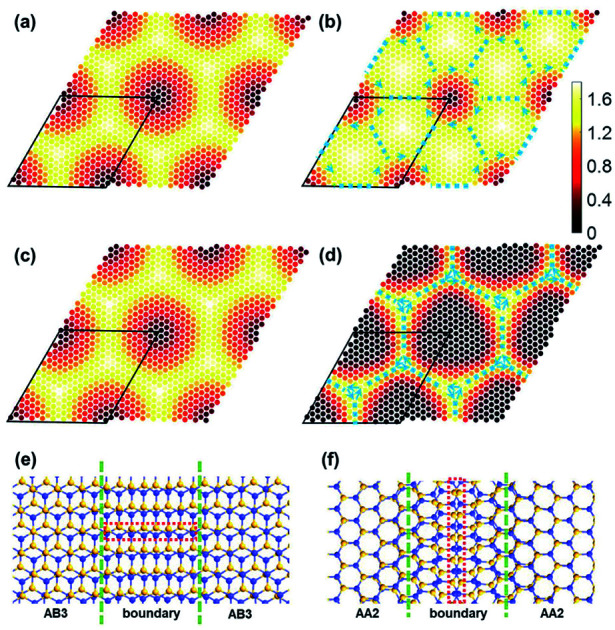

## I. Introduction

Stacking two dimensional (2D) materials vertically by van der Waals (vdW) forces to assemble vdW heterostructures is a promising route to design new functional 2D materials. The moiré superlattice (MSL) arises in vdW heterostructures due to lattice mismatch or misalignment of lattice orientation of constituent 2D materials. Structurally, strain solitons will emerge in the boundaries between adjacent stacking domains in MSLs. Both shear and tensile strain solitons are observed in twisted bilayer graphene (TBG).^1^ The atomic relaxation of MSLs is found to play a dramatic role in the geometric structure of strain solitons.^2–4^ Electronically, exciting phenomena including a Mott-insulating state,^5–8^ unconventional superconductivity,^9,10^ magnetism^11,12^ and topological phases^13^ emerge in TBG. Recently, the experimental technique^14–16^ to manipulate the twist angle in layered 2D materials gained much progress. Except for graphene, other 2D materials with exotic monolayer properties are studied by forming twisted layered systems.^17–19^ Ultra-flat^20,21^ and multi-flat bands,^22^ twist-angle-modulated bandgap,^23^ and a Kagomé lattice^24^ have been revealed.

Monolayer SiC hosts a large direct band gap and an exciton binding energy of up to 2.0 eV, indicating a promising application in optoelectronics and Bose–Einstein condensation.^25–27^ In experiments, monolayer SiC has been successfully fabricated.^28^ However, the effect of twist angle in layered SiC has not been studied so far. To explore the potential excellent properties of layered SiC under interlayer rotation, it is therefore urgent to study the exotic structural and electronic properties of twisted bilayer SiC (TBSC). In this work, the TBSC under a series of typical twist angles is studied using first principles calculations. The atomic structure of TBSC undergoes a dramatic change compared with the rigid shifted bilayer SiC. The triangular and hexagonal stacking domains arise from the expansion of AB3 stacking in α-TBSC and AA2 stacking in β-TBSC. The strain soliton networks with overlapped zigzag atom lines, which can be observed in experiments, separate the adjacent stacking domains. The structural relaxation in TBSC has a large influence on the band edge states of TBSC. The width of the flat bands can be explained by the localization of corresponding band edge states controlled by the moiré potential.

## II. Model and methods

The structural optimization and electronic structure calculations are all performed by the QuantumATK 2019 package.^29^ Double zeta polarized basis is applied to expand the Kohn–Sham orbitals. A density mesh cutoff of 75 Hartree is adopted for the real-space grid. The generalized gradient approximation (GGA) with Perdew–Burke–Ernzerhor (PBE) functional is used to deal with exchange–correlation item. A DFT-D2 vdW correction is adopted to obtain a reasonable interlayer distance in TBSC. The structural optimization is carried out until the residual forces between atoms are less than 0.01 eV Å^−1^. The single Gamma point *k*-sampling is applied to TBSC at twist angle 3.48° (TBSC^3.48^), TBSC^4.41^, TBSC^5.08^ and TBSC^6.01^. 3 × 3 × 1 and 5 × 5 × 1 *k*-samplings are applied to TBSC^7.34^ and TBSC^9.43^ respectively. A 15 Å spacing perpendicular to the SiC surface is adopted to avoid spurious interactions.

**Fig. 1:**
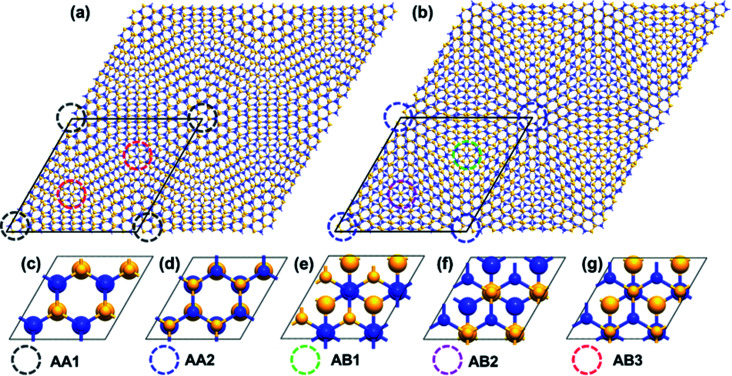
The atomic configuration of (a) α-TBSC^5.08^ and (b) β-TBSC^5.08^. The high-symmetry stacking configurations of bilayer SiC are illustrated in (c)–(g). The colored circles in (a) and (b) label the quasi-high-symmetry stackings emerging in MSL corresponding to the high-symmetry stackings shown in (c)–(g). Note that the radius of atoms in the bottom layers in (c)–(g) is set to be larger than that in the top layer for the convenience of viewing. The yellow and blue balls represent carbon and silicon atoms respectively.

The commensurate lattices of α-TBSC^5.08^ and β-TBSC^5.08^ are shown in Fig. 1a and b, respectively. The local quasi high-symmetry stackings (HSS) emerging in TBSC are highlighted by colored circles. The five HSSs are displayed in Fig. 1c–g. In AA1, the atoms in the two layers are stacked by Si over Si and C over C. In AA2, C over Si and Si over C. In AB1, Si over Si and C over the hollow site. In AB2, C over C and Si over the hollow site. In AB3, half Si over C and half over the hollow site. The TBSC in α phase (α-TBSC) can be interpreted as twisting the bilayer SiC around the geometric center of AA1 HSS (Fig. 1c), while β-TBSC around the geometric center of AA2 HSS (Fig. 1b). Note that AA1 and AB3 HSS emerge in α-TBSC while AA2, AB1 and AB2 HSS emerge in β-TBSC. The commensurate lattice of TBSC is constructed by combining a supercell of lattice constant 
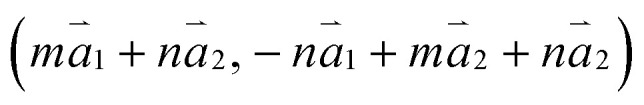
 with that of 
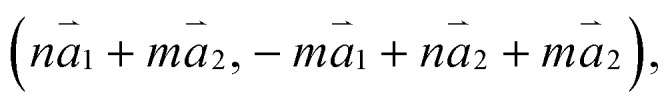
 where 
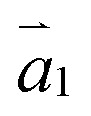
 and 
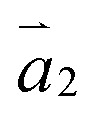
 are lattice vectors of the primitive cell of monolayer SiC. The corresponding twist angle can be expressed as,
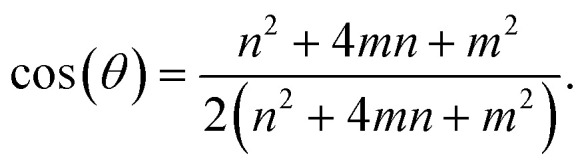


## III. Results and discussions

**Fig. 2:**
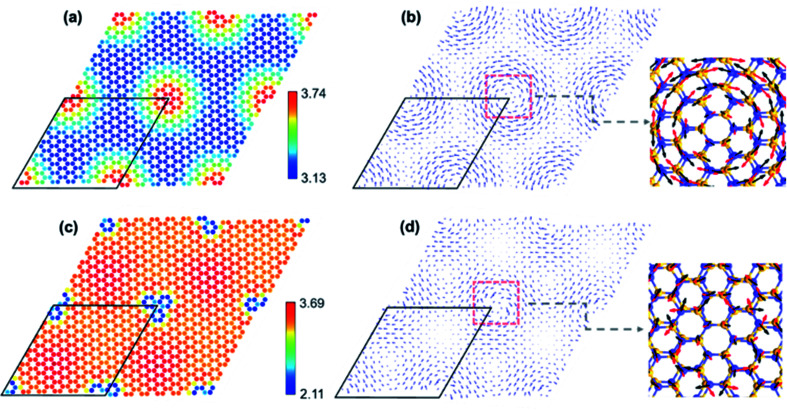
(a) The spatially resolved interlayer distance of α-TBSC^5.08^. (b) The in-plane displacement of the top layer in α-TBSC^5.08^. (c) The spatially resolved interlayer distance of β-TBSC^5.08^. (d) The in-plane displacement of the top layer in β-TBSC^5.08^. The length of the arrows in (b) and (d) is proportional to the distance of in-plane displacement.

The atomic structure of TBSC will undergo a dramatic deformation after relaxation because of the interlayer vdW interaction. The initial structure of TBSC is constructed by placing two supercells of pristine monolayer SiC with a specific twist angle and a constant vertical spacing of 3.3 Å, which we call rigid shifted TBSC (rs-TBSC). Firstly, we present a detailed explanation about the relaxation of α-TBSC^5.08^. After structural optimization, the out-of-plane and in-plane displacements of α-TBSC^5.08^ are plotted in Fig. 2a and b respectively. As shown in Fig. 2a, the local stacking around AA1 HSS (red regions in Fig. 2a) has the maximum interlayer distance of about 3.74 Å, while the local stacking around AB3 HSS (blue regions in Fig. 2a) has a minimum interlayer distance of about 3.13 Å. Interestingly, the local stackings with a short interlayer distance of about 3.13 Å interconnect with each other to construct honeycomb patterns as shown by the quasi-blue regions in Fig. 2a. The in-plane displacements shown in Fig. 2b are calculated by comparing the structurally optimized TBSC (opt-TBSC) with rs-TBSC. A vortex centered at the local stacking with AA1 HSS emerges. Note that the in-plane displacements plotted in Fig. 2b are calculated by comparing the top layer of opt-TBSC and rs-TBSC. The directions of displacements of the bottom layer are inversed. The detailed directions of in-plane displacements in the region labeled by a red dashed rectangle are plotted in a magnified atomic version. The top layer is rotated anti-clockwise around the center of AA1 HSS as labeled by the black arrows, while the bottom layer is rotated clockwise as labeled by the red arrows. Therefore, the interlayer anti-rotation makes the area of quasi-AA1 HSS decrease. Secondly, the atomic relaxation in β-TBSC^5.08^ is analyzed as shown in Fig. 2c and d. The local stacking around AA2 HSS (blue region in Fig. 2c) has the minimum interlayer distance of about 2.11 Å, while the local stacking around AB2 HSS (red regions in Fig. 2c) have a maximum interlayer distance of about 3.69 Å. The in-plane displacement of β-TBSC^5.08^ is shown in Fig. 2d. A vortex centered at the local stacking with AA2 HSS emerges, which is similar to the vortex in α-TBSC^5.08^ as shown in Fig. 2b. However, the rotation direction of the vortex in Fig. 2d is inversed compared with that in Fig. 2b. Therefore, the interlayer mutual-rotation of β-TBSC^5.08^ makes the area of quasi-AA2 HSS increase.

The relaxation of TBSC is the competition between interlayer stacking force and in-plane strain to realize energy minimization. Therefore, the stacking domain with minimum stacking energy will expand as much as possible to minimize the total energy of the system. The stacking energy of five HSSs is listed in Table S1 in ESI.† In α-TBSC^5.08^, the HSSs involved are AA1 and AB3 as shown in Fig. 1a. The stacking energy of AA1 is 1.33 eV, while that of AB3 is 0.67 eV. Thus, the AA1 HSS is energetically unfavorable. The area of quasi-AA1 domains in α-TBSC^5.08^ decreases after relaxation. In β-TBSC^5.08^, the HSSs involved are AA2, AB1 and AB2 as shown in Fig. 1b. The stacking energy of AA2, AB1 and AB2 are 0 eV, 0.90 eV and 1.31 eV, respectively. Thus, the AA2 HSS is the most stable stacking energetically. The area of quasi-AA2 domains in β-TBSC^5.08^ increases after relaxation.

**Fig. 3:**
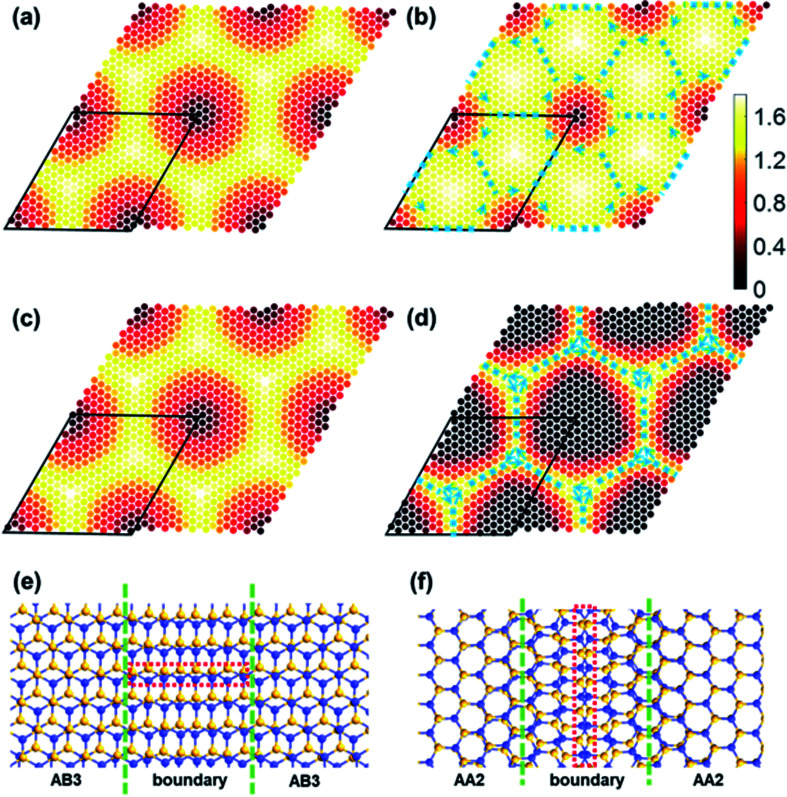
The modulus of the order parameter in real space. (a) and (b) Display rigid shifted and structurally optimized α-TBSC^3.48^ respectively, while (c) and (d) display rigid shifted and structurally optimized β-TBSC^3.48^ respectively. The blue dashed lines in (b) and (d) represent the soliton occurring at stacking domain boundaries. The arrows label the directions of the order parameter 
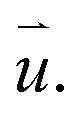
 (e) and (f) Present the atomic structure of shear solitons in structurally optimized α-TBSC^3.48^ and β-TBSC^3.48^ respectively.

Strain solitons will emerge in MSLs of TBSC. To investigate the evolution of strain solitons under structural relaxation, an order parameter 
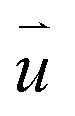
 is defined. In α-TBSC, the order parameter 
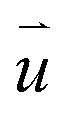
 is defined as the shortest vector of the interlayer shift to convert the local stackings into AA1 HSS. The 
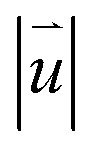
 of rs-α-TBSC^3.48^ and opt-α-TBSC^3.48^ in real space is shown in Fig. 3a and b, respectively. The quasi-black and quasi-white regions in Fig. 3a which are centered at AA1 HSS and AB3 HSS in rs-TBSC represent the low and high value of 
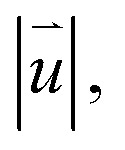
 respectively. In the line connecting the AA1 and AB3 HSS, a smooth transition takes place from 
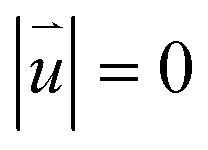
 to 
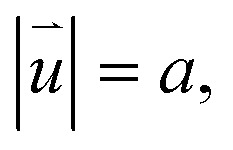
 where *a* is the Si–C bond length. After structure relaxation, the quasi-black region reduces, while the quasi-white region expands. This contraction and expansion correspond to the same variation trend of the AA1 and AB3 HSS. Following the expansion of the quasi-white region with a high value 
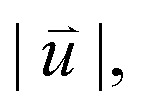
 triangle domains of AB3 HSS emerge. The boundary of adjacent domains is strain soliton, which is labeled by the blue dashed line in Fig. 3b. The direction of 
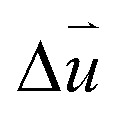
 across the strain soliton is parallel to the line of strain soliton. Therefore, this kind of strain soliton is a shear-strain soliton.^1^ The direction of the order parameter 
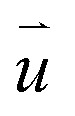
 rotates 2π in a circle (blue arrows in Fig. 3b) centered at AA1 HSS, indicating this region is a topological defect.

In β-TBSC, the order parameter 
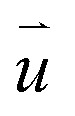
 is defined as the shortest vector of the interlayer shift to convert the local stacking into AA2 HSS. The 
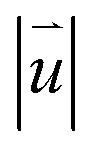
 of rs-β-TBSC^3.48^ and opt-β-TBSC^3.48^ distributed in real space is shown in Fig. 3c and d, respectively. The real space distribution of 
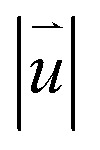
 in rs-β-TBSC^3.48^ is the same as that of rs-α-TBSC^3.48^. However, the 
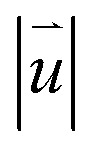
 of opt-β-TBSC^3.48^ exhibits a dramatic different behavior compared with that of opt-α-TBSC^3.48^. As the AA2 HSS is most energetically stable in β-TBSC, the expansion of AA2 HSS induces a large area of the quasi-black region with a low value of 
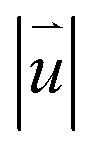
 as shown in Fig. 3d. The quasi-AA2 HSS domains relax into hexagons separated by the quasi-yellow strain soliton boundaries labeled as blue dashed lines as shown in Fig. 3d. The strain soliton is a shear-strain soliton, as the direction of 
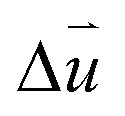
 across the strain soliton is parallel to the line of strain soliton. At the six vertexes of each hexagon, small vortexes of 
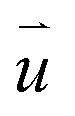
 emerge. Among these six vortexes, three vortexes correspond to AB1 HSS, and the other three corresponds to AB2 HSS. Around the center of the quasi-black hexagon, the order parameter 
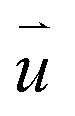
 rotates by 2π, which is the same as that in α-TBSC.

The triangle and hexagon stain soliton networks in α-TBSC and β-TBSC can be observed in the experiment by infrared nanoscopy^30^ or transmission electron microscopy.^1^ The atomic configuration of strain solitons emerging in TBSC is shown in Fig. 3e and f. In α-TBSC, the soliton is the boundary between AB3 domains, while the soliton is the boundary between AA2 domains in β-TBSC. The soliton consists of nearly overlapped lines of zigzag atoms as labeled by the red dashed rectangles in Fig. 3e and f. The overlapped lines can be imaged by STEM.^1^ Interestingly, the overlapped lines of zigzag atoms in α-TBSC are perpendicular to the solitons, while that in β-TBSC is parallel to the solitons. The unique shape of strain solitons in TBSC may have a dramatic influence on the electronic transmission^31^ and find application in MSL heterostructures.^32^

**Fig. 4:**
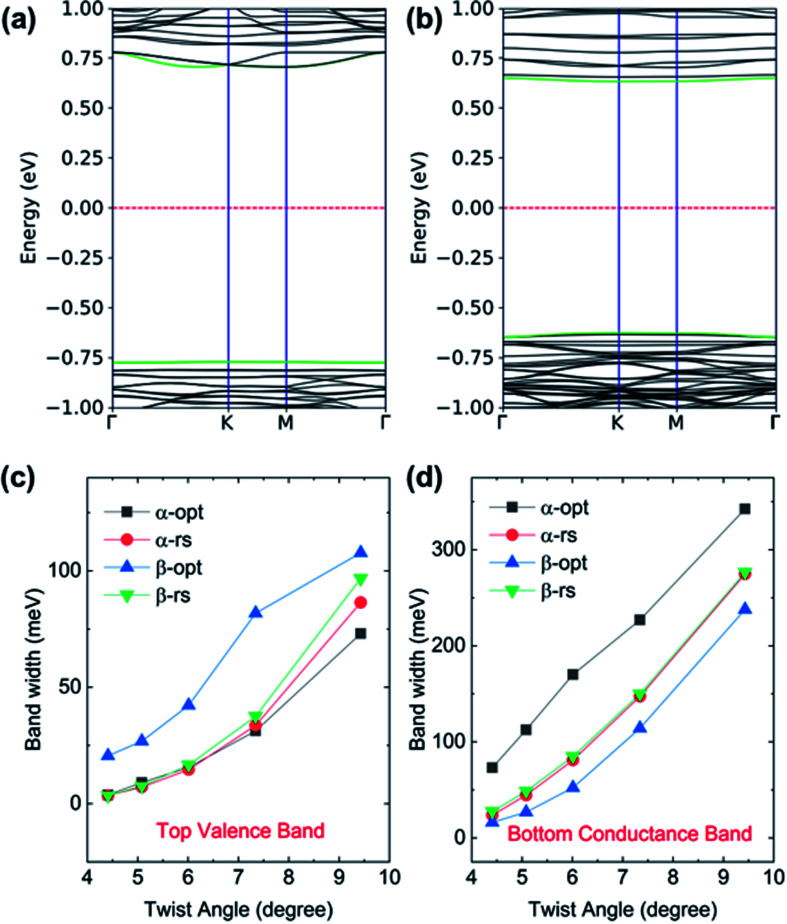
The band structure of (a) α-TBSC^4.41^ and (b) β-TBSC^4.41^. (c) and (d) The width of the top valence band and bottom conduction band of TBSC which is structurally optimized (opt) or rigid shifted (rs) near the band gap at five twist angles studied in this work, respectively.

The electronic structure of TBSC will be profoundly influenced by twist angle and structural relaxation. The band structure of opt-α-TBSC^4.41^ and opt-β-TBSC^4.41^ are plotted in Fig. 4a and b. The top valence band (TVB) and bottom conduction band (BCB) are highlighted in green color. Flat bands emerge which are especially obvious for the valence band of α-TBSC^4.41^ and the conduction band of β-TBSC^4.41^. To investigate the effect of twist angle and structure relaxation on the flat band width, the band width of TVB and BCB of structurally optimized and rigid shifted α-TBSC and β-TBSC as a function of twist angle is plotted in Fig. 4c and d. Firstly, a common trend of decreasing band width with decreasing twist angle is observed. The ultra-flat band can be obtained at low twist angles. For example, the width of the top valence band of opt-α-TBSC^4.41^ is about 4 meV. This ultraflat band indicates a potential Mott-insulator phase at proper carrier density. The large ratio U/W represents the possibility of a Mott-insulator phase, where U is the on-site Coulomb interaction and W is the band width of flat band. The typical band width in twisted bilayer graphene at 1.08° is 12 meV. Therefore, the several meV band width of twist bilayer SiC may possess the similar Mott-insulating state at proper carrier density. The size *a*_m_ of MSL can be written as a function of twist angle *θ* as 
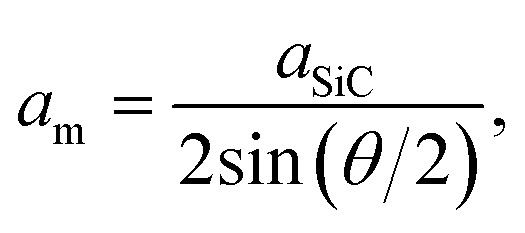
 where *a*_SiC_ is the lattice constant of the primitive cell of monolayer SiC. Therefore, the spatial confinement effect will be enhanced with decreasing twist angle as the size of MSL increases. The spatial confinement hinders the interaction among the localized states in the flat band. Thus, the flat band width will decrease into a flat line in the limit of ideal isolation of localized states without any interactions. Secondly, the structure relaxation has a profound effect on the band width as shown in Fig. 4c and d. Interestingly, the structure relaxation can either increase or decrease the flat band width. For example, the width of TVB in opt-β-TBSC^4.41^ is larger than that in rs-β-TBSC^4.41^. However, the width of BCB in opt-β-TBSC^4.41^ is smaller than that in rs-β-TBSC^4.41^.

**Fig. 5:**
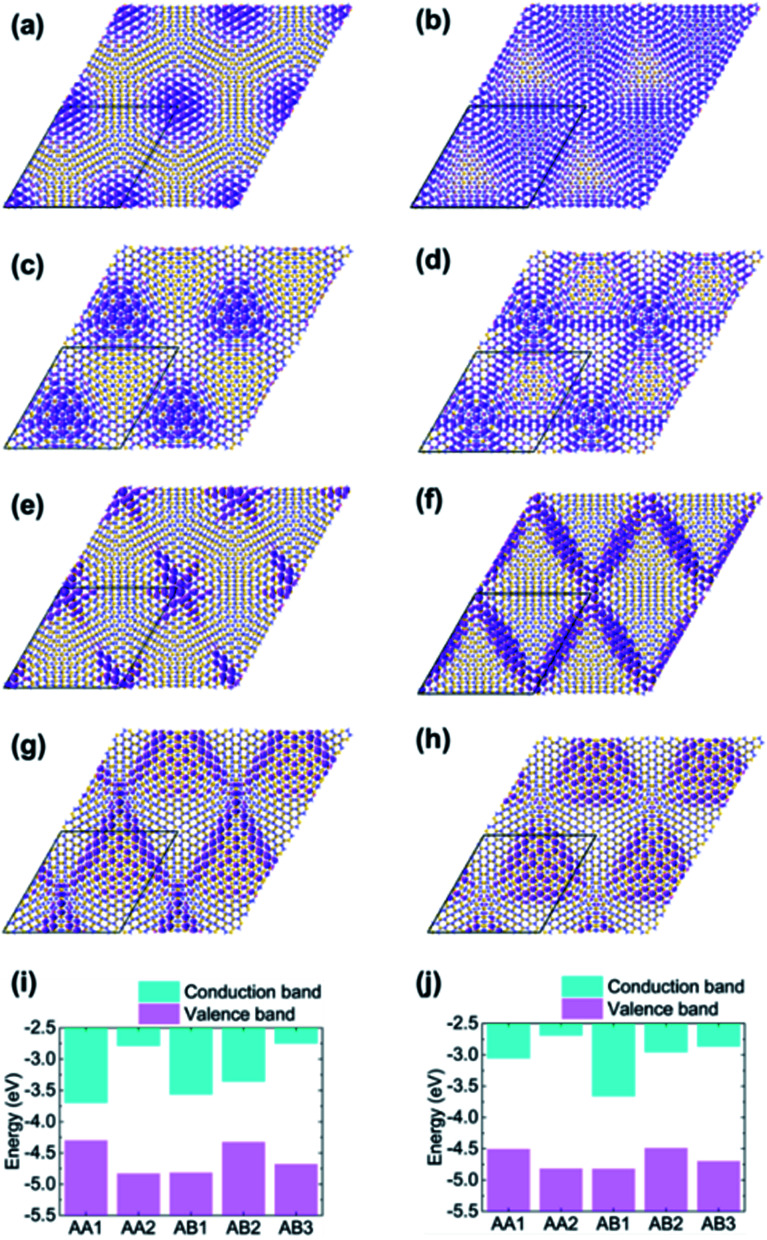
The real-space wavefunction-modulus with an isovalue of 0.02 Å^−1.5^ corresponding to (a) VBM of rigid shifted α-TBSC^4.41^, (b) VBM of structurally optimized α-TBSC^4.41^, (c) VBM of rigid shifted β-TBSC^4.41^, (d) VBM of structurally optimized β-TBSC^4.41^, (e) CBM of rigid shifted α-TBSC^4.41^, (f) CBM of structurally optimized α-TBSC^4.41^, (g) CBM of rigid shifted β-TBSC^4.41^, (h) CBM of structurally optimized β-TBSC^4.41^ (i) the band edge of bilayer SiC at high-symmetry stackings with rigid shift interlayer distance of 3.3 Å. (j) The band edge of bilayer SiC at high-symmetry stackings with interlayer distance extracted from structure optimized α-TBSC^4.41^ and β-TBSC^4.41^.

The dependence of flat band width on structure relaxation can be interpreted by the localization of corresponding states modulated by moiré potential. The modulus of wavefunction according to the band edge states is plotted in Fig. 5a–h. Firstly, the localization pattern of rs-TBSC is analyzed as shown in Fig. 5a, c, e and g. In Fig. 5a, the wavefunction of valence band maximum (VBM) is regularly localized in the region with quasi-AA1 HSS. This localization pattern can be well explained by the band edge alignment of HSSs with a fixed interlayer distance of 3.3 Å as shown in Fig. 5i, which is calculated separately by aligning the band energies to the vacuum level. Note that the energy alignment of HSS band edges will not change by using higher order hybridized HSE functional (Fig. S1 in ESI†). The general rule is HSS with the highest VBM will dominate the contribution to the VBM of TBSC, while HSS with the lowest conduction band minimum (CBM) will dominate the contribution to the CBM of TBSC. The wavefunction of VBM or CBM shall localize in the HSSs making the most contribution. The HSSs involved in α-TBSC are AA1 and AB3. The VBM of AA1 is higher than that of AB3 as shown in Fig. 5i. Therefore, the wavefunction of VBM in rs-α-TBSC^4.41^ is localized in the AA1 HSS as shown in Fig. 5a. To check the validity of this rule, we check that the wavefunction of VBM in rigid shifted β-TBSC^4.41^ is localized in AB2 HSSs. As the HSSs involved in β-TBSC are AA2, AB1 and AB2, the highest VBM belongs to AB2, which confirms the rule. In addition, the localization of CBM state in AA1 in α-TBSC^4.41^ as shown in Fig. 5e can be explained by the lowest CBM energy of AA1, among the involved AA1 and AB3 HSSs (Fig. 5i). The localization of CBM state in AB1 in β-TBSC^4.41^ as shown in Fig. 5g can be explained by the lowest CBM energy of AB1, among the involved AA2, AB1 and AB2 HSSs (Fig. 5i).

The localization pattern of the band edge state changes because of the energy alignment renormalization induced by structure relaxation. The energy alignment order of band edges of HSSs in relaxed TBSC is not changed as shown in Fig. 5j. However, the band edges of some specific local structures become comparable with that of HSSs. Therefore, by comparing the wavefunction of rs-TBSC and opt-TBSC, the localization patterns of opt-TBSC delocalize into the local structures with matched energies as shown in Fig. 5b, d and f. The delocalization of wavefunction can explain the band width increasement phenomena as shown in Fig. 4c and d. Note that the wavefunction can also become more localized after structure relaxation as shown in Fig. 5h, if the size of the local structure with the proper band edge energy decreases. This makes the band width of the bottom conduction band of β-TBSC^4.41^ flater as shown in Fig. 4d.

In conclusion, we study the structural and electronic properties of α-TBSC and β-TBSC with a series of twist angle from 3.48° to 9.43°. The structure of the TBSC is fully relaxed in the scheme of DFT calculation. The local stackings in TBSC undergo expansion or contraction because of the energy minimization requirement. An order parameter is adopted to describe the stacking order transition in the TBSC. The triangular and hexagonal stacking domains emerge in α-TBSC and β-TBSC, respectively. The shear solitons with overlapped zigzag atom lines form triangle and hexagon networks to separate the low energy stacking domains. The stacking domains and shear solitons can be observed in experiments by infrared nanoscopy or transmission electron microscopy. Flat bands emerge in the band edge near Fermi level. The width of the flat band depends sensitively on structure relaxation. The moiré-potential-modulated localization pattern of state in the flat band is found to control the flat band width. The moiré-pattern-induced band structure could lead to the moiré exciton^33,34^ and Mott-insulator phase.^35^ The localization of band edge states may find application in quantum dot and quantum computation. Therefore, the moiré-pattern-induced structural and electronic properties of TBSC are promising for application in nanoscale electronic and optic devices.

## Conflicts of interest

There are no conflicts to declare.

## Supplementary Material

RA-011-D1RA02139G-s001
