# Supplementary material for: Multi-shaped strain soliton networks and moiré-potential-modulated band edge states in twisted bilayer SiC
Source: RSC Adv. 2021 Jul 12;11(39):24366–73. doi: 10.1039/d1ra02139g (PMC9036811; doi:10.1039/d1ra02139g)
Supplement: RA-011-D1RA02139G-s001 [file RA-011-D1RA02139G-s001.pdf]

Electronic Supplementary Material (ESI) for RSC Advances.
This journal is © The Royal Society of Chemistry 2021

Supplementary materials for

Multi-shaped strain soliton networks and moiré-potential-modulated band edge states in twisted bilayer SiC

Dawei Kang,*[,1] Zheng-Wei Zuo,[1] Zhaowu Wang[1,2] and Weiwei Ju[1]

[1]*School of Physics and Engineering, Henan University of Science and Technology, Luoyang 471023, China*

[2]*National Laboratory of Solid State Microstructures, Nanjing University, Nanjing 210093, China*

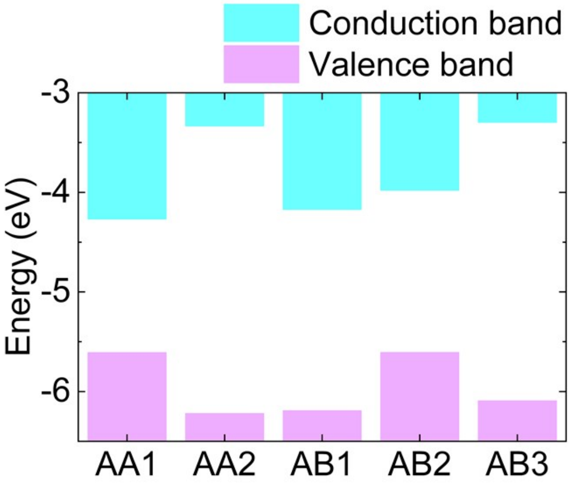

Fig.1. The band edge of bilayer SiC calculated by HSE functionals at high-symmetry stackings with rigid shift interlayer distance of 3.3 Å.

The stacking energies of bilayer SiC at high symmetry stackings are calculated as $E_{stack} = 2E_{monolayer} - E_{bilayer}$.

Table 1. The interlayer distance of bilayer SiC at high-symmetry stackings (HSS)

HSS	AA1	AA2	AB1	AB2	AB3
Interlayer distance (Å)	3.77	2.21	3.24	3.65	3.15
Total energy (eV)	1.33	0	0.90	1.31	0.67

* kdwsdu@163.com

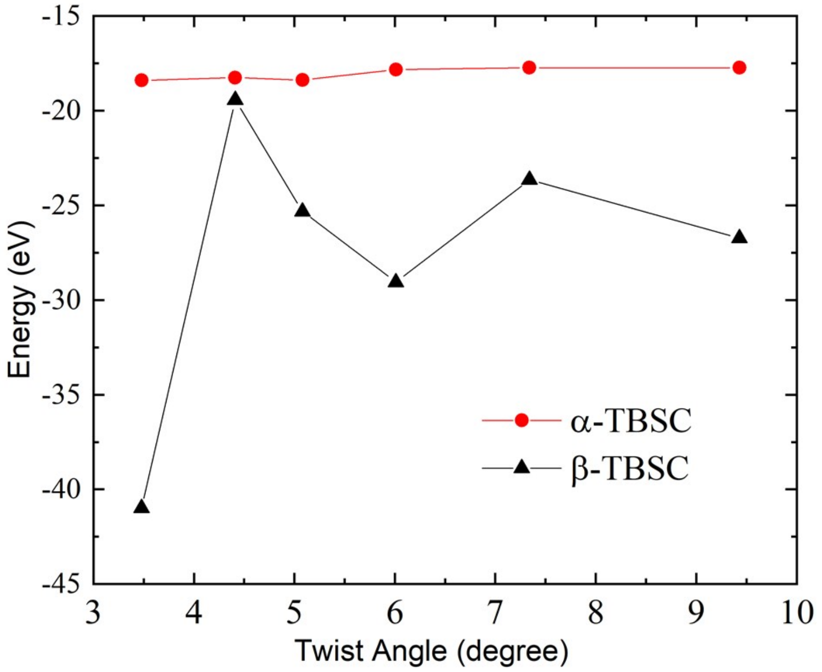

Fig. 2. The cohesive energy of TBSC as a function of twist angle.

Table 1. The (m,n) and number of atoms in TBSC studied in this work

Twist Angle	(m,n)	Number of atoms
3.48	(9,10)	1084
4.41	(7,8)	676
5.08	(6,7)	508
6.01	(5,6)	364
7.34	(4,5)	244
9.43	(3,4)	148
